# Perception of the operating agents about the Brazilian National School Feeding Program

**DOI:** 10.11606/S1518-8787.2019053000597

**Published:** 2019-03-26

**Authors:** Daniele Mendonça Ferreira, Roseane Moreira Sampaio Barbosa, Nathália Corrêa Finizola, Daniele da Silva Bastos Soares, Patrícia Henriques, Silvia Pereira, Clarice Soares Carvalhosa, Ana Beatriz Franco Sena Siqueira, Patricia Camacho Dias

**Affiliations:** I Universidade Federal Fluminense. Faculdade de Nutrição Emília de Jesus Ferreiro. Departamento de Nutrição Social. Niterói, RJ, Brasil

**Keywords:** School feeding, Nutrition Programs and Policies, Institutional Management Teams, Nutrition, Public Health, Alimentação escolar, Programas e Políticas de Nutrição e Alimentação, Equipes de Administração Institucional, Nutrição em Saúde Pública

## Abstract

**OBJECTIVE:**

Identify the perception operating agents have on the Brazilian National School Feeding Program.

**METHODS:**

This is an observational, cross-sectional and quali-quantitative study developed in non-probability and convenience sampling selected in an event promoted by the National Fund for Educational Development in 2015 in Rio de Janeiro. Data were collected through questions related to pre-defined categories concerning the National School Feeding Program. The questionnaire was answered by 43 nutritionists, 41 members of the School Feeding Board, and 16 school feeding administrators from 38 cities of Rio de Janeiro. The narrative analysis was based on benchmarks of cognitive analysis of public policies. The association among variables was investigated with chi-square test, being calculated the power for association testing.

**RESULTS:**

The perception of the implementation of the National School Feeding Program has been characterized by some challenges: 1) low number of nutritionists to meet the demand from schools; 2) low adhesion to the public call for the purchase of family farming products due to bureaucratic difficulties and insufficient local food production; 3) reduced coverage of food and nutritional measures due to the restriction of human, material and financial resources; and 4) limitation of the participation of the School Feeding Board due to insufficient training and transport for regular visits. The adequacy of the number of nutritionists showed statistically significant association with the purchase of family farming products (p = 0.002; power = 99%) and with the food and nutritional education activities (p = 0.021; power = 79%).

**CONCLUSIONS:**

The results indicate the need for employment of nutritionist in sufficient numbers to meet the demands of the National School Feeding Program, investment in educational activities of healthy eating in schools, training of the School Feeding Board, greater availability of vehicles for school visits and assistance to family farmers in order to facilitate their participation in programs of institutional purchases and encourage the diversification of production.

## RESUMO

**OBJETIVO:**

Identificar a percepção de agentes operadores sobre o Programa Nacional de Alimentação Escolar.

**MÉTODOS:**

Trata-se de um estudo observacional, transversal e quali-quantitativo desenvolvido em amostra não probabilística e selecionada por conveniência em um evento promovido pelo Fundo Nacional de Desenvolvimento da Educação em 2015 no Rio de Janeiro. Os dados foram coletados com o auxílio de um questionário cujas questões se relacionavam a categorias pré-definidas relacionadas ao Programa Nacional de Alimentação Escolar. O questionário foi respondido por 43 nutricionistas, 41 membros do Conselho de Alimentação Escolar e 16 gestores da alimentação escolar de 38 municípios do Rio de Janeiro. A análise das narrativas foi baseada em referenciais de análise cognitiva de políticas públicas. A associação entre as variáveis foi investigada com o teste qui-quadrado *,* sendo calculado o poder do teste das associações.

**RESULTADOS:**

A percepção da execução do Programa Nacional de Alimentação Escolar foi caracterizada por alguns desafios: 1) baixo quantitativo de nutricionistas para atender à demanda das escolas; 2) baixa adesão à chamada pública para compra de produtos da agricultura familiar por dificuldades burocráticas e insuficiência de produção local de alimentos; 3) reduzida abrangência das ações de educação alimentar e nutricional pela restrição dos recursos humanos, materiais e financeiros; e 4) limitação na atuação do Conselho de Alimentação Escolar por oferta insuficiente de capacitação e de transporte para as visitas regulares. A adequação do quantitativo de nutricionistas mostrou associação estatisticamente significante com a compra de produtos da agricultura familiar (p = 0,002; poder = 99%) e com as atividades de educação alimentar e nutricional (p = 0,021; poder = 79%).

**CONCLUSÕES:**

Os resultados indicam a necessidade de contratação de nutricionistas em número suficiente para atender às demandas do Programa Nacional de Alimentação Escolar, investimento em atividades educativas de alimentação saudável nas escolas, capacitação do Conselho de Alimentação Escolar, maior disponibilidade de veículos para visitas escolares e assistência aos agricultores familiares para facilitar a participação em programas de compras institucionais e oportunizar a diversificação de produção.

## INTRODUCTION

The National School Feeding Program (PNAE) is one of the largest and oldest programs in the world and, in Brazil, is part of the Food and Nutrition Security Policy ^1^ . Its main objectives are related to the provision of meals that ensure nutritional needs during the stay of the students in the school and the formation of healthy food habits ^2^ . Since its implementation in 1955, the legal devices governing the program have presented technical and operational advances through reviews and upgrade^3–5^ aiming to guarantee the human right to an adequate diet (DHAA). These changes involve the inclusion of a nutritionist as a responsible technician (RT), the social control of the implementation of the program by the School Feeding Board (CAE) and the purchase of family farming products, with an investment of at least 30% of the financial allocation made by the National Fund for Educational Development (FNDE) ^3^ ^,^ ^6^ .

Considering PNAE is one of the most important health promotion programs in the country, a fact evidenced by its contribution to the formation of healthy eating habits of students from primary public education ^5^ , it is essential that its principles and guidelines are implemented fully by the agents involved. However, several Brazilian cities have problems with its execution, such as the low percentage of purchases of family farming foodstuffs ^7^ ^,^ ^8^ , failure to comply with the responsibilities of CAE as social control ^9^ , the erroneous use by executing entities (EE) of financial allocations carried out by FNDE ^10^ , the planning of menus without considering the seasonality and diversity of regional food ^11^ , and the insufficient number of nutritionists ^12^ .

Such limitations should be better understood to facilitate the proposition of strategies that contribute to overcoming the challenges. However, few studies explore the context in which the measures envisaged by the program are developed, expressing the origin of these obstacles superficially. Thus, this study aimed to identify the perception operating agents of PNAE have about the fulfilment of its primary legal parameters.

## METHODS

### Ethical Aspects

The study is in line with the Resolution 466 of December 12, 2012. This study was approved by the Research Ethics Committee of the Universidade Federal Fluminense (CAAE 3620.0.000.258-10).

All the participants signed an informed consent form after being informed of the procedures in this study. The questions were answered in writing without personal identification or the city. Only the participant’s position was identified for data analysis.

### Design and Sample

This is a descriptive and observational cross-sectional study with a quali-quantitative approach developed in a non-probability and convenience sampling. The operating agents of PNAE present at the event “Technical Meeting of the National School Feeding Program” ^a^ , promoted by FNDE in 2015 in the metropolitan region of Rio de Janeiro, were invited to participate.

The sample size calculation was carried out with the pwr Pack of the R program version 3.4.3 (R Core Team, Austria). Considering the factors and respective levels of the semi-structured questionnaire, the power of test of 80%, and the significance level of 5%, 85 participants are needed (4x3: 6 degrees of freedom) to observe a medium to large effect size (w = 0.4).

A total of 189 operating agents of PNAE from 58 cities participated in the event, being excluded those living outside the state of Rio de Janeiro (n = 5). One hundred individuals agreed to participate in the research, including nutritionists (n = 43), members of CAE (n = 41) and school feeding administrators of (n = 16) from 38 (41.3%) of the 92 cities of the state of Rio de Janeiro. Among the cities represented in this study, 33 (86.8%) are predominantly urban, while 5 cities (13.2%) are rural ^13^ . The distribution of agents by city comprehended: one city with nine agents, two cities with six agents, one city with five agents, four cities with four agents, 11 cities with three agents, six cities with two agents, and 13 cities with only one agent.

### Procedures

The information was collected through a semi-structured questionnaire created by the researchers based on the categories of analysis predefined in this study. The questions addressed the following items related to the implementation of PNAE in the cities of Rio de Janeiro: adequacy of the number of nutritionists in the program; percentage of purchase and foodstuffs from family farming; main difficulties found in the implementation of PNAE; development of educational activities to promote a healthy eating habit at schools; and the activity of CAE in the city.

### Qualitative Data Analysis

We used a contents and narrative analysis ^14^ as a tool to identify the main ideas and perceptions the subjects have about the operationalization of PNAE in the cities studied. To this end, the following steps were followed:

1. Pre-exploration of the material: skimming of all the answers contained in the questionnaire to seize and organize in a non-systematized way the main ideas and perceptions that provided initial evidence for a more systematized presentation of the data in the following steps;
2. Ex ante categorization: use of predefined categories based on the implementation of PNAE: role of nutritionists; purchase of family farming products; activity of CAE in the city; food and nutritional education measures; several difficulties in implementing the PNAE.
3. Selection of subcategories of analysis: guided by the questions, corresponding to items related to the implementation of PNAE. The subcategories of analysis were identified from text clippings by an inductive process, considering the explicit message and the non-apparent meanings of the context. The grouping of units of analysis is based on the repetition of contents common to most respondents and on the implicit relevance, when the topic was considered important for the study, but it was not repeated by the respondents.

This whole process was conducted along with the researchers to avoid perceived bias and to provide a different view of the categories proposed.

The narrative analysis was based on the perception of the subjects participating in the operationalization of PNAE in cities of the state of Rio de Janeiro. Thus, the program was considered a social practice ^15^ and analyzed based on benchmarks of the field of cognitive analysis of public policies ^16^ .

### Statistical Analysis

Data were tabulated in contingency tables and analyzed in the statistical program SPSS version 21 (IBM, USA). The variables were described by absolute and relative frequency (n, %). The association between the categorical variables was investigated through the chi-square test (χ^2^). For 2 x 2 tables, the Fisher’s exact test was used. The power of test (1-β) was calculated with the R program version 3.4.3 (R Core Team, Austria) and the pwr pack. A significance level (α) of 5% was adopted.

## RESULTS

The Box presents qualitative synthesis of the perception operating agents of PNAE have about the key challenges faced in the implementation of the program in their cities. *A priori* , five categories were defined related to the main items of PNAE. *A posteriori* , fourteen subcategories were identified by reflecting the difficulties expressed in the contents reported. The following categories and subcategories include: role of nutritionists (assignments, quantitative adequacy, workload and employment), purchase of family farming products (acquisition of aptitude certificate for the National Program for Strengthening Family Farming, local production and foodstuffs purchased), activity of CAE in the city (transport, training of members and participation), food and nutritional education measures (insufficient number of nutritionists, breadth of educational measures and financial resources), and difficulties in implementing the PNAE (financial allocation and excessive administrative work for the nutritionist).

Table 1 shows data on the implementation of the items proposed by PNAE according to the reports of operating agents. Most nutritionists (n = 39; 90.7%) and members of CAE (n = 34; 75.6%) consider the number of professionals insufficient to perform all the activities assigned by PNAE (p = 0.008; power = 80%). This opinion is related to the high demand of school units with a large number of students ( Box, item A1 ), hindering the assistance and implementation of monitoring tasks of the nutritional status of students and development of projects to promote healthy eating and nutritional education activities. The overload and the urgency of bureaucratic and administrative tasks were also cited as a barrier to the implementation of technical assignments ( Box, item E2 ).

**Table 1:** Characteristics of the implementation of the National School Feeding Program according to reports of the operating agents from 38 cities of Rio de Janeiro.

Characteristic	Sample			Total	p*; power

	School feeding administrator	Members of CAE	Nutritionists		
			
	n = 12	n = 45	n = 43	n = 100	
Insufficient number of nutritionists					**0.008; 0.80**
Yes	5 (26.3%)	11 (57.9%)	3 (15.8%)	19 (19.0%)	
No	6 (7.6%)	34 (43.0%)	39 (49.4%)	79 (79.0%)	
No answer	1 (50.0%)	0 (0.0%)	1 (50.0%)	2 (2.0%)	
Purchase of family farming products					0.080; 0.51
Over 30%	8 (17.0%)	17 (36.2%)	22 (46.8%)	47 (47.0%)	
Below 30%	1 (5.0%)	13 (65.0%)	6 (30.0%)	20 (20.0%)	
No answer	3 (9.0%)	15 (45.5%)	15 (45.5%)	33 (33.0%)	
Purchase of organics					0.390; 0.21
Yes	0 (0.0%)	5 (45.5%)	6 (54.5%)	11 (11.0%)	
No	11 (14.2%)	33 (42.9%)	33 (42.9%)	77 (77.0%)	
No answer	1 (8.4%)	7 (58.3%)	4 (33.3%)	12 (12.0%)	
Food and nutritional education activities					0.289; 0.27
Yes	11 (13.4%)	34 (41.5%)	37 (45.1%)	82 (82.0%)	
No	1 (6.3%)	10 (62.5%)	5 (31.3%)	16 (16.0%)	
No answer	0 (0.0%)	1 (50.0%)	1 (50.0%)	2 (2.0%)	
Activity of CAE					**0.009; 0.86**
Good	6 (9.1%)	36 (54.5%)	24 (36.4%)	66 (66.0%)	
Regular	6 (23.1%)	9 (34.6%)	11 (42.3%)	26 (26.0%)	
Bad	0 (0.0%)	0 (0.0%)	6 (100.0%)	6 (6.0%)	
No answer	0 (0.0%)	0 (0.0%)	2 (100.0%)	2 (2.0%)	

CAE: School Feeding Board

Values are presented in absolute and relative frequency (%).

Values with statistical significance are presented in bold.

* Chi-square test for association among categorical variables.

**Box:** Categories of analysis on the difficulties of implementing the parameters recommended by the National School Feeding Program (PNAE), according to reports from its operating agents.

Categories	Subcategories	Narratives
A. Role of nutritionists	1. Attributions:	“Work overload is inevitable, given the many tasks involved in the planning, execution and accountability to PNAE.” (School feeding administrator) “Activities are very complex, especially monitoring visits to school units, considering the demands for the fulfillment of PNAE.” (Member of CAE)
	2. Quantitative adequacy	“For the resolution no. 465/2010 of CFN (Federal Council of Nutritionists), there should be 7 [nutritionists] (RT + 6 TF); however, we have 1 RT and 1 TF.” (Nutritionist) “We are not within the framework established by CFN.” (Nutritionist)
	3. Workload	“I work only 20 hours in PNAE and there are no other nutritionists. It’s impossible to fulfill all activities required by Resolution No. 465.” (Nutritionist) “A nutritionist with workload of 20 hours and working at 14 schools struggles to give all necessary support to the schools.” (Nutritionist)
	4. Employment relationship	“There’s only one civil servant, the ones hired, although they have competence, can’t get autonomy.” (Member of the CAE)
B. Purchase of family farming products	1. Acquisition of the PRONAF aptitude certificate	“Many farmers have no interest in acquiring the DAP, as they have to pay fees or taxes, and [they] have good marketing of their products at the fairs.” (Nutritionist) “We can’t reach a higher percentage due to the number of DAP.” (Member of CAE)
	2. Local production	“The demand in the city is higher than the number and variety of foodstuffs offered by the local cooperative.” (Nutritionist) “Family farming production is insufficient. Farmers in other cities are not interested in participating in the public call.” (Member of CAE)
C. Activity of the CAE in the city	1. Transport	“Cars are not always available for school visits and monitoring of the school feeding.” (Member of CAE) “There is no transport for regular visits.” (Member of CAE)
	2. Qualification of members	“The advisers are not trained and informed about their responsibilities.” (School feeding administrator) “I think the members of the board should be trained, because there is a lack of knowledge.” (School feeding administrator)
	3. Participation	“Not all members participate actively.” (School feeding administrator) “There is no engagement of CAE with the activities that should be developed – visits, management monitoring.” (Nutritionist)
D. Food and nutritional education activities	1. Insufficient number of nutritionists	“Schools eventually develop some activities, but the participation of the nutritionist in these activities is difficult.” (Nutritionist) “Very few [educational activities] are performed, because [there is] just one nutritionist for everything.” (Member of CAE)
	2. Scope of educational activities	“A lot of initiatives were developed by the civil society (boards) and committed technicians, but we did not reach the ideal [(public policy)].” (Member of CAE) “When something happens, it is developed by some schools in an isolated way.” (Member of CAE) “Arranging ‘meetings’ with the lunch ladies.” (Nutritionist) “School activities for the lunch ladies were performed (“Cozinha Brasil”) in partnership with Sesi of Rio de Janeiro.” (Nutritionist)
	3. Financial resources	“[...] and there could be financial allocation for nutritional education activities.” (Nutritionist)
E. Several difficulties in the implementation of PNAE	1. Financial allocation	“The greatest difficulty refers to the funds for purchase of food. On the other hand, we have to meet the nutritional needs.” (Nutritionist) “The allocation value is too small to meet the nutritional needs of the students.” (Nutritionist)
	2. Excessive administrative work for the nutritionist	“Create a nutrition sector with professionals to carry out administrative work.” (Nutritionist) “As the nutritionists perform administrative activities for a long time, it is difficult to conduct activities on all schools to promote healthy eating.” (Nutritionist)

CAE: School Feeding Board; SESI: *Serviço Social da Indústria* ; PRONAF: National Program for Strengthening Family Farming; DAP: PRONAF Aptitude Certificate; RT: responsible technician; TF: technical framework

The purchase of family farming products over 30% of the resources provided by FNDE was reported by 70.1% (n = 47) of respondents, while the purchase of organics had a frequency of 12.5% (n = 11). However, these variables did not show statistically significant association with the operating agents of PNAE (p > 0.05). Foods commonly purchased from family farming come *in natura* form, such as lettuce, tomatoes, cassava, yams, parsley and chives, cabbage, pumpkin, wild cabbage, banana, orange, pineapple, sweet potato, carrot, beet, persimmon, okra, eggs, honey, and milk. Analyzing the answers per city, 55.3% (n = 21) were buying over 30%, while 10.5% (n = 4) had not acquired foodstuffs yet for being in a public call process. The main justification presented for the purchase of family farming products was the enforcement of legislation. Although they met the requirement, 21.0% (n = 8) of cities reported difficulties due to the lack of the aptitude certificate for the National Program for Strengthening Family Farming (PRONAF) by farmers ( Box, item B1 ) and for the lack of local food production (Table, item B2).

The existence of educational activities to promote healthy eating habits in schools was reported by 83.7% (n = 82) of the respondents. Training through workshops with the lunch ladies were the main activities carried out ( Box, item D2 ). In a lower frequency, group dynamics with students and lectures with the parents promoted by nutritionists, teachers and educators were also included in nutritional education. However, the frequency of these activities was not mentioned. Among the main criteria established as gridlock for the implementation of educational activities are the difficulties with human resources, due to the insufficient number of nutritionists and other professionals ( Box, item D1 ), and the availability of financial and material resources to develop workshops routinely (Table, item D3).

Most respondents (n = 66; 67.3%) classified the activity of CAE in the cities as good and significant (p = 0.009; power = 86%); the members of the board (n = 36; 80%) classified their own work as good. One of the measures developed includes technical visits and supervision of schools, participation in meetings, conversations with students about the acceptance of menus and monitoring of the implementation of PNAE resources. Regular and bad qualifications were mostly attributed to the lack of training for board members and transport for regular visits ( Box, items C2 and C1, respectively ).

Table 2 shows the statistically significant association of quantitative adequacy of nutritionists in PNAE with the purchase of family farming products (p = 0.002; power = 99%) and with the food and nutritional education activities (EAN) (p = 0.021; power = 79%), indicating that the cities struggling to fulfill the legislation parameters are those with insufficient number of nutritionists, according to the participants. The opinion about the quality of the activity of CAE did not show statistically significant association with the purchase of organic foods (p = 0.729; power = 10%) and family farming (p = 0.273; power = 28%), nor with the activities of EAN (p = 0.905; power = 7%).

**Table 2:** Association between the adequacy of the number of nutritionists and quality of the activity of the School Feeding Board (CAE) and the parameters recommended in the National School Feeding Program, according to the operating agents.

Variable	Purchase of family farming products		p*; power	Purchase of organics		p*; power	Food and nutritional education activities		p*; power
		
	< 30%	> 30%		Yes	No		Yes	No	
Adequacy of the number of nutritionists									
Yes	0 (0.0%)	15 (100.0%)	**0.002; 0.99**	3 (17.6%)	14 (82.4%)	0.374; 0.11	19 (100.0%)	0 (0.0%)	**0.021; 0.79**
No	20 (38.5%)	32 (61.5%)		8 (11.6%)	61 (88.4%)		61 (79.2%)	16 (20.8%)	
Activity of CAE									
Good	16 (36.4%)	28 (63.6%)	0.273; 0.28	8 (13.8%)	50 (86.2%)	0.729; 0.10	55 (84.6%)	10 (15.4%)	0.905; 0.07
Regular	3 (17.6%)	14 (82.4%)		2 (8.7%)	21 (91.3%)		21 (80.8%)	5 (19.2%)	
Bad	1 (16.7%)	5 (83.3%)		1 (20.0%)	4 (80.0%)		5 (83.3%)	1 (16.7%)	

Values are presented in absolute and relative frequency (%).

Values with statistical significance are presented in bold.

* Chi-square test for association among categorical variables.

Isolating the quantitative answers of the operating agents of PNAE from the same city (25 cities with more than one agent), few showed differences among themselves. Regarding the adequacy of the number of nutritionists and purchase of organic foods, only 8% (n = 2) of the cities presented divergent answers. As to the purchase from family farming, 16% (n = 4) of cities showed disagreement. The questions with more differences were those related to the implementation of activities of EAN (n = 6; 26% of cities) and CAE (n = 5; 20.8% of cities). This observation is important to identify whether the answers reflect the reality of each city.

## DISCUSSION

The integrated narrative analysis, based on different segments of subjects who participate directly in the operationalization of PNAE in the cities, indicates as the main strategic questions for the quality of the program aspects related to the institutional structure, the articulation with family farmers, the financial contribution, and social participation.

About the role of nutritionists, almost all of them reported an insufficient number of professionals, which is a possible limitation for the fulfilment of the tasks established in the program, generating workload. The optimal number of nutritionists to participate in the program is referenced in the Resolution 26/2013 of FNDE5 ^5^ , which follows the numeric parameters for employment of professionals by execution unit of PNAE established in Resolution 465/2010 of the Federal Council of Nutritionists (CFN) ^4^ . According to this resolution, all execution entity must have a nutrition technician responsible for the program, regardless of the number of students. Above 501 students, there must also be a nutritionist within the technical framework (TF), increasing the number of nutritionists as the number of students rise. Reports of the existence of one nutritionist for each 5,000 students were observed, scenario where there should be a nutritionist RT and three nutritionists of TF ^4^ .

Two other important matters mentioned by nutritionists are the weekly workload of 20 hours and the difference of employment relationship among professionals, which contributes to the reduction of autonomy and turnover of those working under CLT regime. Given this scenario, the monitoring and implementation of the PNAE guidelines are compromised, hindering the control of the nutritional status of students, food and nutrition education activities and the supervision of the entire production process of meals, since the acquisition of food until the distribution in order to meet the principles of food and nutrition security and ensure hygienic sanitary conditions ^11^ ^,^ ^17^ .

As to the participation of family farming in school meals, the minimum percentage of acquisition became mandatory as of January 2010 ^3^ , favoring the sustainability, reduction of rural exodus and increase in job offers with local development ^6^ . In this study, most cities (55.3%) reported the purchase of family farming products over 30% of federal financial resources. However, this percentage was lower than that found by Ferigollo et al. ^18^ (71.2%) in a study carried out in the cities of Rio Grande do Sul. According to Saraiva et al. ^6^ , the South stands out in the production of family farming and the internal supply of food, which can justify its successful experience. Some cities of Rio de Janeiro still face difficulties, which, according to the reports, refer mainly to the lack of the PRONAF (DAP) aptitude certificate to compete for the public call process and the insufficient local production to meet the demand of school meals. The DAP is used to identify and qualify the family units of agrarian production and to give access to PRONAF credit lines and other public purchase policies of the federal government, such as the Food Acquisition Program (PAA) and the PNAE. According to the operating agents of PNAE, the main obstacle is related to bureaucratic issues, such as the existence of farmers who do not declare or declare a reduced portion of sales through invoices, hindering the proof of agricultural income required for the issuance of DAF.

Another difficulty reported about the purchase of family farming products is linked to the lack of compliance with the demand for school meals to local production. Some cities, especially those with more urban-rural characteristics, present insufficient production in quantity and variety of foodstuffs. Although the legislation allows the purchase from farmers in neighboring cities, some reports showed that they are not interested in selling due to the logistics of foodstuff transportation to schools. Saraiva et al. ^6^ , in a study on the subject in the five Brazilian regions, attributed the non-compliance with the legislation to the lack of proper planning of menus and to the inadequate estimate of the demand of food needed, stressing the importance of integrating buyers and farmers. Articulations are needed between farmers and school feeding administrators, being implemented in the form of technical and logistical support to facilitate the integration of producers into the markets and the access to the public call ^19^ . In this study, we observed that the insufficient number of nutritionists in the program, according to the participants, was associated with an insufficient percentage of purchases. Thus, the adequacy of the number of these professionals is an important factor for the development of menus suitable for the local culture, for the strengthening of the relationship with family farming and, finally, for the enforcement of the legislation.

In a study carried out in cities of the southern region and published in 2015 ^9^ , we observed support for the participation of the small farmer in PNAE. However, only one of the three cities assessed reached the minimum of 30%. The success of the participation of family farming in PNAE goes beyond the support for the farmer, requiring the valuation and adequate training of the nutritionist working in this process. The approach of topics that go beyond the health field, such as school feeding and food and nutrition security, which cover issues related to the production, supply, marketing, access, and food consumption, occupy an incipient position in the curriculum of nutrition courses in public and private higher education institutions in the country ^20^ .

Saraiva et al. ^6^ stressed the need to insert this purchasing process in various governmental and public policies and highlight as a key factor the planning of strategies capable to address these challenges. Among the strategies, the authors describe the adequacy of institutional structures as fundamental and indicate as examples the technical advisory for farmers, adequacy of logistics and storage infrastructure, diagnosis of the local agricultural reality and the creation of spaces for discussion and planning with farmers, public administrators, and schools.

As provided for in article 19 of the Resolution June 26, 2013 ^5^ , the purchase of organic foods must be a priority in PNAE. The low percentage reported on the acquisition of organic foods was not related to the number of nutritionists of the program, which may be due to the difficulty in establishing a partnership with unions of producers of organic foods and planning or to the search for the best price, because its cost superior to that of conventional foods hinders the inclusion in school menus. However, according to the current legislation, organic or agroecological products can be purchased for up to a value 30% higher compared with the prices established for conventional products ^5^ .

The Organic School Feeding Program, from Santa Catarina, receives financial resources from the state ^21^ . Silva and Souza ^22^ , studying the offer and demand for organic food for school meals, observed that in 2010 only 17.7% of the cities in Santa Catarina purchased this type of food. A total of 42% of the nutritionists reported having difficulties in the acquisition and use of organic foods in PNAE, such as: lack of products on the market, resistance of lunch ladies due to the foods’ appearance, prices and lack of organic certification.

Regarding the social control of the program, the study showed that some cities lack visits from the CAE to the schools, claiming lack of transport. Other difficulties reported refer to the lack of training for its members and adhesion to the activities assigned by the legal devices. According to legislation ^5^ , the EE must offer the necessary infrastructure for the transport of the members of the board, and the failure to comply with this obligation impairs the monitoring of the guidelines of the program. Similar results were found by Souza ^23^ , who observed a malfunction of CAE in the cities of Minas Gerais and Espírito Santo, highlighting the inadequate working conditions of advisers and the ignorance of the guidelines and regiments of the program. To promote training spaces for members of the board is a way of strengthening its role as a supervisory body and the appropriate application of financial resources on school meals.

In this study, the participants classify the activity of CAE in the cities between good and regular, a perception that is not significantly associated with purchases of family farming products and organic food, nor with carrying out activities of EAN. It is important to consider that this is the perception of the participants and not an assessment or analysis of the role of the entity. Some studies have found that, in cities where CAE held about 20 meetings a year, there was the hindrance of school feeding outsourcing and closer monitoring of the development and acceptance of the menus by students ^10^ ^,^ ^24^ , as well as the demand for the recruitment of nutritionists to the program, enhancing the practical influence of boards on the improvement of PNAE ^7^ and on the support for healthy eating.

Among the PNAE guidelines, the need for inserting food and nutritional education in the teaching-learning process has gained prominence. For the development of healthy eating habits, it is not enough to offer a menu; educational measures are also needed for a better understanding of the students, who are going through the cognitive development process ^25^ . Such curricular insertion has been developed through topics related to food and nutrition security, comprising food, nutrition, and practices for healthy living ^3^ . We highlight the importance of EAN, from the food and nutrition security approach, in the expansion of the senses assigned to healthy eating in a perspective of DHAA among the school agents ^26^ .

According to FNDE, EAN is regarded as “the set of formative measures, of continuous, permanent, transdisciplinary, multidisciplinary, intersectoral, and multi-professional practice, aiming to encourage the voluntary adoption of healthy practices and better food choices that collaborate to a learning experience, to the student’s health state and to the individual’s quality of life” ^27^ . The study showed scarce food and nutrition educational activities with the school community as a whole, and the most affected cities were those with insufficient nutritionists, according to the participants. The absence of the theme in the education network does not contribute to the effectiveness of PNAE, as the practice must be combined with the theory for a significant construction of knowledge about healthy eating.

Considering the large number of schools for each nutritionist in EAN, we suggest investing in the training of agents of the school community who are involved with the program, such as teachers, food handlers, principals and parents, in order to share experiences in food and nutrition and contribute to building a healthy eating environment. Gabriel et al. ^9^ , in a study that assessed the municipal management of PNAE in the three capitals of the south region of Brazil, noted the absence of pedagogical activities in most of the cities studied. Only one capital carried out food and nutrition education projects involving all municipal education network. One of the capitals provided training in EAN with some teachers, but not implemented the training evaluation methodology. Two capitals inserted healthy nutrition into the school curriculum, but that insertion was considered incipient.

Despite the activity of EAN being mandatory and the role of the nutritionist in PNAE ^2^ ^,^ ^4^ ^,^ ^5^ , Mello et al. ^17^ , in a study conducted in northeastern cities, noted that only 33% of the nutritionists interviewed performed them with frequency. The lack of stimulus, low salaries, and devaluation of the professional were indicated as possible factors for this low frequency. Voos and Schuch ^28^ , studying the role of the nutritionist in PNAE in the state of Rio Grande do Sul, observed that the main reasons reported for not performing these activities were the low number of nutritionists to meet the demand and the lack of support and interest of the authorities of the city to solve the problem.

The results of this study should be interpreted considering its advantages and limitations. First, the participation of different operating agents of PNAE (nutritionists, administrators and members of CAE) of approximately 40% of the cities in the state suggests a moderate representation of Rio de Janeiro. The inclusion of other cities can contribute to socially different perceptions and should be considered in future studies. Second, the perception operating agents have about the implementation of PNAE was investigated so reality could be portrayed and interpreted in accordance with the position the professional occupies in the organizational structure of the program. Although perception and implementation may differ, the perception that the program is not contemplated in any way justifies a reflection upon its execution. The identification of perceptions in this study reveals the challenges faced in the implementation of PNAE, resulting in the construction of new perspectives to be directed to the solution of problems.

## CONCLUSIONS

The perceptions of operating agents were based on the role of the nutritionist in the program, on the purchase of family farming products and organics, on the activities of EAN and CAE as a mechanism of social control. The insufficient number of nutritionists and the inadequate employment relationship were considered limiting factors for the accomplishment of all the tasks assigned to the professional. The insufficient number of nutritionists who work in the program showed significant association with the reduced percentage of purchase of family farming products and EAN activity in the school environment. The activities, in turn, were scarce when directed to the promotion of healthy eating habits with the school community. Although most respondents classified the activity of CAE as good, the lack of training courses for advisers, the lack of transport for school visits and the lack of engagement of all members in the execution of their duties in the board are some of the current difficulties.

Thus, based on the main difficulties experienced by operating agents of the program, we suggest as strategic measures for the strengthening of PNAE: (1) employment of nutritionists in sufficient numbers to meet the demands of PNAE, (2) investment in educational activities to promote healthy eating habits in the school environment, (3) training of members of CAE and availability of vehicles for school visits and (4) technical support, within the municipal management framework, for organic and family farmers, in order to facilitate the organization and participation in programs of institutional purchases and encourage the diversification of production.

## Footnotes

a Encontro Técnico do Programa Nacional de Alimentação Escolar, 23 set 2015; Niterói, RJ [cited 2019 Feb 1]. Available from: http://www.uff.br/?q=events/i-encontro-tecnico-do-programa-nacional-de-alimentacao-escolar
